# Exploring the epidemiology of suicide attempts: Risk modeling in Kermanshah—Iran

**DOI:** 10.3389/fpubh.2022.924907

**Published:** 2022-08-23

**Authors:** Nahid Khademi, Alireza Zangeneh, Arash Ziapour, Shahram Saeidi, Raziyeh Teimouri, Komali Yenneti, Shahrzad Moghadam, Ali Almasi, Shirin Zardui Golanbari

**Affiliations:** ^1^Department of Disease Prevention and Control, Kermanshah University of Medical Sciences, Kermanshah, Iran; ^2^Social Development and Health Promotion Research Center, Health Institute, Kermanshah University of Medical Sciences, Kermanshah, Iran; ^3^Cardiovascular Research Center, Health Institute, Kermanshah University of Medical Sciences, Kermanshah, Iran; ^4^UniSA Creative, University of South Australia, Adelaide, SA, Australia; ^5^School of Architecture and the Built Environment, Faculty of Science and Engineering, University of Wolverhampton, Wolverhampton, United Kingdom; ^6^Geography and Urban Planning, University of Zanjan, Zanjan, Iran; ^7^Department of Medical Records, Kermanshah University of Medical Sciences, Kermanshah, Iran

**Keywords:** suicide attempt, GIS, Kermanshah, Iran, spatial patterns, public health

## Abstract

**Background:**

Suicide attempt is a serious global public health issue. The patterns of suicide may vary depending on the individual characteristics, methods, causes, and the geographical area-also socio-cultural context that determine it. This study identifies the spatial patterns of suicide attempts in Kermanshah province, Iran.

**Method:**

The sample size of this cross-sectional study is 18,331 people (7234 males and 11097 females) who attempted suicide in Kermanshah province between 2006 and 2014. Data was collected from the records of patients referred to the emergency department of hospitals in Kermanshah and analyzed using tests of Mean Center, Standard Distance, and Average Nearest Neighbor.

**Results:**

The results of the mean center and standard distance tests show that drug overdose, poisoning with toxins and pesticides, and chemicals mostly were used in the central areas of Kermanshah province. The mean center of suicide attempts by self-immolation, hanging and firearms was in the western parts of the province, while the suicide attempts with narcotic drug were concentrated in the eastern regions of the province. Out of the 18,331 cases, 74% attempted suicide with drug overdose, 13% with toxins and pesticides, 0.59% with chemicals, 4% with fire, 1% by self-immolation, 1% by hanging, 0.16% with firearms and 0.7% with cold weapons. The spatial pattern of all suicide attempts in Kermanshah was clustered (Z-score < −2.58).

**Conclusion:**

The results of this study show that the methods of suicide attempt vary with geographical areas in the province. Therefore, it is suggested that planning tailored to the geographical location can reduce suicide attempts in Kermanshah.

## Introduction

Suicide is one of the major public health problems across the world (1) and has recently imposed huge costs on communities and health care systems (2). Suicide attempt is a manifestation of one's suffering and has many negative effects on family, friends, and society (3, 4). Studies have shown that 20–70% of those who have committed suicide have attempted suicide at least once in the past. In general, suicide attempts are 8–25 times more common than suicides (5). Investigating methods of suicide attempt is a very important aspect of suicidal behaviors because the use of violent methods is directly related to the outcome of suicide (6). Existing literature suggests that suicide methods are different in various parts of the world. For example, the most common method of suicide in the United States (both sexes), Colombia (men), Switzerland (men), and Uruguay (both sexes) is using a firearm. Jumping from a height is more common in countries such as Hong Kong, Luxembourg and Malta. Hanging is most commonly reported in continental Europe (Denmark, France, Germany, Iceland, Netherlands and Portugal), the Americas (Argentina, Brazil, Canada and Venezuela), Asia (Japan, South Korea and Thailand), South Africa, and Australia. Although self-immolation is a rare phenomenon in developed countries, it has been reported as one of the three most common methods of suicide in countries such as Pakistan, Sri Lanka, Iran and India (7–12). The common methods of suicide in north-eastern Iran are self-harm, hanging and jumping from a height. In south-western Iran, methods of self-immolation and self-harm with sharp tools have been reported. Hanging and self-immolation methods are more evident in southern Iran. In western Iran, methods of hanging, self-mutilation, self-immolation and jumping from a height are common (7, 13, 14).

Suicide patterns vary according to individual characteristics, methods, causes, and geographical areas; even in a specific geographical area the pattern may be different (15). Therefore, suicide reduction planning of one country or geographical region with specific culture and socio-economic status may not be beneficial for other regions (16). In this regard, geographic information system (GIS) can be used as a suitable tool to identify critical and risk areas, and the correct public health management policies and decisions (17, 18). In addition, GIS can be used in suicide centers to monitor early detection and intervention measures (19). More specifically, correct understanding of the spatial distribution of suicide methods can help to plan and evaluate suicide prevention efforts (20).

A large body of literature in Kermanshah province has investigated the determinants or prevalence of suicidal behaviors (21–24), but the spatiality of suicides has been neglected. This is while the results of studies over the past decade indicate an increasing suicide trend in Kermanshah province, and other studies have emphasized the need for a study on suicide methods (25). The present study aims to determine the spatial patterns of methods for suicide attempt using GIS in Kermanshah province.

## Materials and methods

### Study design

The sample of this cross-sectional study was 18,331 people (7,234 males and 11,097 females) who attempted suicide in Kermanshah province during 2006 to 2014 by drug overdose, consuming toxins and pesticides, chemical poisoning, self-immolation, narcotic drug poisoning, hanging, firearms, cold weapons, jumping from height, and other methods. Data was collected from the records of patients referred to the emergency departments of various hospitals in Kermanshah province.

### Statistical analyses

The Arc/GIS Desktop 10.6 was used with the necessary tools for analyzing data and displaying high quality results. This software is a professional geographic information system that offers a variety of fantastical tools. It applied for using descriptive data and spatial information. In this research the ArcGIS (Esri, New York, NY, USA) was used.

Patient data was processed in ArcGIS10.6 software. Mean Center and Standard Distance tests, as well as Average Nearest Neighbor index were used to examine the spatial pattern of suicide attempts in the province. The Spatial Statistics Tools for Mapping Clusters include the nearest neighbor index, mean center and standard distance tests. The mean center test is the average of the longitude and latitude coordinates of all features in the study area, which is calculated to follow the changes in spatial distribution of features and also to compare them. The mean center was calculated by the following equation: (18, 26).


$$\begin{array}{l} X=\sum_{i=1}^{N}\frac{Xi}{N}Y=\sum_{i=1}^{N}\frac{Yi}{N} \end{array}$$

The standard distance test is a method to measure the concentration or scattering of geographic features around the center and was calculated by the following formula (17, 18, 26):


$$\begin{array}{l} \sqrt{\frac{\sum_{i=1}^{n}\left(x_{i}-\bar{X}\right)}{n}+\frac{\sum_{i=1}^{n}{\left(y_{i}-\bar{Y}\right)}^{2}}{n}} \end{array}$$

The nearest neighbor index was based on measuring the distance between suicide attempt methods and the nearest neighbor, and was used to determine the convergence and divergence of suicide attempt methods. This analysis determines whether or not the distribution of suicide attempt methods is random and also specifies the type of distribution pattern. The nearest neighbor index shows the ratio of the observed average distance to the expected distance. The expected distance in this method was obtained as a result of Z quantity analysis. If this value is between 1.96 to −1.96, there is no significant difference between the observed distribution and random distribution. Else, the distribution will be clustered or uniform.

The nearest neighbor index is obtained from the following relation:


$$\begin{array}{l} ANN=\frac{\overset{-}{D_{o}}}{\overset{-}{D_{E}}} \end{array}$$

In which ${\overset{-}{D}}_{o}$ is the average distance between each of the suicide attempts to the nearest neighbor, obtained from the following relation:


$$\begin{array}{l} \bar{D_{o}}=\frac{\sum_{i=1}^{n}di}{n} \end{array}$$

The average of expected distance for the index obtained is a random pattern.

In this equation, ${\overset{-}{D}}_{E}$ is the distance between the index *i* and its nearest neighbor, *n* is the sum of the indexes, and *A* is equal to the total area studied.

### Ethics approval and consent to participate

The principal investigators conducted this study in accordance with the Helsinki Declaration and followed the ethical standards for the scientific research procedures. The Ethics Committee of the Kermanshah University Medical Sciences under number IR.KUMS.REC.1397.1064 approved this study. All participants were informed about the study and only those providing written informed consent were enrolled in the study.

## Results

During the study period, a total of 18,331 suicide attempts had occurred of which, 13,699 cases (74.73%) attempted with drug overdose, 2,531 cases (13.8%) with toxins and pesticides, 109 cases (0.59%) with chemicals, 845 cases by self-immolation (4.6%), 258 cases with narcotics (1.4%), 200 cases by hanging (1.09%), 31 cases by firearms (0.16%), 130 cases with self-mutilation (0.7%), and 3 cases with jumping from height, while 316 cases were categorized as others methods and 209 cases were unknown (Table 1).

**Table 1:** Demographic characteristics of suicide attempts in Kermanshah province (2006–2014).

**Variables**		**Drug overdose**	**Poison**	**Chemical poisoning**	**Fire**	**Drug poisoning**	**Hanging**	**Firearms**	**Cold weapon**	**Jumping from height**	**Other methods**	**Missing data**	**Total**
		***N* (%)**	***N* (%)**	***N* (%)**	***N* (%)**	***N* (%)**	***N* (%)**	***N* (%)**	***N* (%)**	***N* (%)**	***N* (%)**	***N* (%)**	***N* (%)**
Gender	Male	5,532	930	50	133	124	121	23	89	1	131	100	7,234
		(40.38)	(36.74)	(45.87)	(15.74)	(48.06)	(60.5)	(74.19)	(68.46)	(33.3)	(41.45)	(47.85)	(100)
	Female	8,167	1,601	59	712	134	79	8	41	2	185	109	11,097
		(59.62)	(63.26)	(54.13)	(84.26)	(51.94)	(39.5)	(25.81)	(31.54)	(6.66)	(58.54)	(52.15)	(100)
	Total	13,699	2,531	109	845	258	200	31	130	3	316	209	18,331
Age group	<15	510	96	7	31	5	7	0	3	0	8	9	676
		(3.72)	(3.79)	(6.6)	(3.67)	(1.94)	(3.5)	(0)	(2.31)		(2.48)	(4.31)	(3.69)
	16–25	6,833	1,067	62	320	106	72	10	74	3	80	126	8,753
		(49.88)	(42.16)	(58.49)	(37.87)	(41.08)	(36)	(32.27)	(56.92)	(100)	(24.84)	(60.29)	(47.75)
	26–35	1,916	362	26	177	44	37	1	25	0	24	33	2,645
		(13.99)	(14.3)	(24.53)	(20.95)	(17.05)	(18.5)	(3.22)	(19.23)		(7.46)	(15.79)	(14.43)
	36–45	444	170	5	77	17	6	2	6	0	16	14	757
		(3.24)	(6.72)	(4.72)	(9.11)	(6.59)	(3)	(6.45)	(4.61)	(0)	(4.97)	(6.69)	(4.13)
	46–55	165	82	1	38	10	9	0	3	0	7	8	323
		(1.2)	(3.24)	(0.94)	(4.5)	(3.87)	(4.5)	(0)	(2.31)	(0)	(2.17)	(3.83)	(1.76)
	56–65	73	52	2	15	4	1	1	0	0	2	3	153
		(0.53)	(2.05)	(1.89)	(1.77)	(1.55)	(0.5)	(3.22)			(0.63)	(1.43)	(0.83)
	66+	44	44	1	16	1	1	0	0	0	63	7	177
		(0.32)	(1.74)	(0.94)	(1.9)	(0.39)	(0.5)				(19.56)	(3.35)	(0.97)
	Missing	3,714	658	2	171	71	67	17	19	0	119	9	4,847
		(27.11)	(25.99)	(1.89)	(20.24)	(27.52)	(33.5)	(54.84)	(14.62)		(37.89)	(4.31)	(26.44)
	Total	13,699	2,531	109	845	258	200	31	130	3	316	209	18,331
Marital status	Single	8,003	1,262	57	319	108	113	25	90	2	161	115	10,255
		(58.42)	(49.86)	(52.29)	(37.75)	(41.86)	(56.5)	(80.65)	(69.23)	(66.67)	(50.95)	(55.02)	(55.94)
	Marital	5,307	1,166	43	476	133	76	6	36	1	144	83	7,471
		(38.74)	(46.07)	(39.45)	(56.33)	(51.55)	(38)	(19.35)	(27.69)	(33.33)	(45.57)	(39.71)	(40.76)
	Divorced/Widow	302	83	7	45	11	8	0	4	0	9	2	471
		(2.2)	(3.28)	(6.42)	(5.32)	(4.26)	(4)		(3.08)		(2.85)	(0.96)	(2.57)
	Missing	87	20	2	5	6	3	0	0	0	2	9	134
		(0.63)	(0.79)	(1.83)	(0.59)	(2.32)	(1.5)				(0.63)	(4.31)	(0.73)
	Total	13,699	2,531	109	845	258	200	31	130	3	316	209	18,331
		(100)	(100)	(100)	(100)	(100)	(100)	(100)	(100)	(100)	(100)	(100)	(100)
Educational status	Illiterate	699	417	10	195	27	23	6	5	0	36	21	1,439
		(5.1)	(16.47)	(9.17)	(23.08)	(10.46)	(11.5)	(19.35)	(3.85)		(11.39)	(10.05)	(7.85)
	Low education	5,767	1,206	44	415	102	114	12	46	0	159	76	7,941
		(42.1)	(47.65)	(40.37)	(49.11)	(39.53)	(57)	(38.71)	(35.38)		(50.32)	(36.36)	(43.32)
	Media education	5,472	664	38	182	90	46	7	59	1	109	53	6,721
		(39.94)	(26.23)	(34.86)	(21.54)	(34.88)	(23)	(22.58)	(45.39)	(33.33)	(34.49)	(25.36)	(36.66)
	High education	1,188	114	3	28	14	5	1	10	2	9	15	1,389
		(8.67)	(4.5)	(2.75)	(3.31)	(5.43)	(2.5)	(3.23)	(7.69)	(66.67)	(2.85)	(7.18)	(7.85)
	Missing	573	130	14	25	25	12	5	10	0	3	44	841
		(4.18)	(5.14)	(12.84)	(2.96)	(9.69)	(6)	(16.13)	(7.69)		(0.95)	(21.05)	(4.59)
	Total	13,699	2,531	109	845	258	200	31	130	3	316	209	18,331
Result suicide attempt	Unsuccessful	13,455	2,462	104	434	253	127	15	126	2	307	192	17,477
		(98.22)	(97.27)	(95.41)	(51.36)	(98.06)	(63.5)	(48.39)	(96.92)	(66.67)	(97.15)	(91.87)	(95.34)
	Successful	73	34	3	388	2	71	16	2	1	6	2	598
		(0.53)	(1.34)	(2.75)	(45.92)	(0.77)	(35.5)	(51.61)	(1.54)	(33.33)	(1.9)	(0.96)	(3.26)
	Missing	171	35	2	23	3	2	0	2	0	3	15	256
		(1.25)	(1.38)	(1.83)	(2.72)	(1.16)	(1)		(1.54)		(0.95)	(7.17)	(1.4)
	Total	13,699	2,531	109	845	258	200	31	130	3	316	209	18,331
Place of residence	Rural	3,331	1,103	28	368	61	75	20	34	0	103	53	5,176
		(24.31)	(43.58)	(25.69)	(43.55)	(23.64)	(37.5)	(64.51)	(26.15)		(32.59)	(25.36)	(28.24)
	Urban	10,368	1,428	81	477	197	125	11	96	3	213	156	13,155
		(75.69)	(56.42)	(74.31)	(56.45)	(76.36)	(62.5)	(35.4)	(73.85)	(100)	(67.41)	(74.64)	(71.76)
	Total	13,699	2,531	109	845	258	200	31	130	3	316	209	18,331

The results of methods according to gender show that suicide attempts with drug overdose (59.62%), toxins and pesticides (63.26%), self-immolation (84.26%), narcotics (51.94%) and chemicals (54.13%) were more common in women while firearms (74.19%), self-mutilation (68.46%), and hanging (60.5%) were more evident in men. Young people between 16-25 years had the highest attempted suicide rate among all the age groups. In single people, methods of drug overdose (58.42%), chemicals (52.29%), hanging (56.5%), firearms (80.65%) and self-mutilation (69.23%) were more common, while in married people, self-immolation (56.33%) and narcotics (51.55%) were evident methods. People with low education had the highest number of suicide attempts in all methods except the use of self-mutilation (45.39%) which was more common in people with high school education. In all the methods, the number of unsuccessful attempts was higher and the percentage of fatal acts was higher only in those using the firearms (51.61%). Further, the analysis of suicide attempts by location show that only the use of firearms (64.51%) was more common in rural areas.

The results of the mean center and standard distance tests show that drug overdose, poisoning with toxins and pesticides, and chemicals mostly were used in the central areas of province. The mean center of suicide attempts by self-immolation, hanging and firearms was in the western parts of the province. Suicide attempts with narcotic drug poisoning were concentrated in the eastern regions of Kermanshah province. On the other hand, the results of suicide attempts through self-mutilation show that the standard deviation of this method was larger than other methods and covered more areas of Kermanshah province. Probably, this situation has different reasons such as emotional and behavioral problems, especially with symptoms of depression and anxiety. As a result, the standard deviation observed to require further investigation.

**Figure 1:**
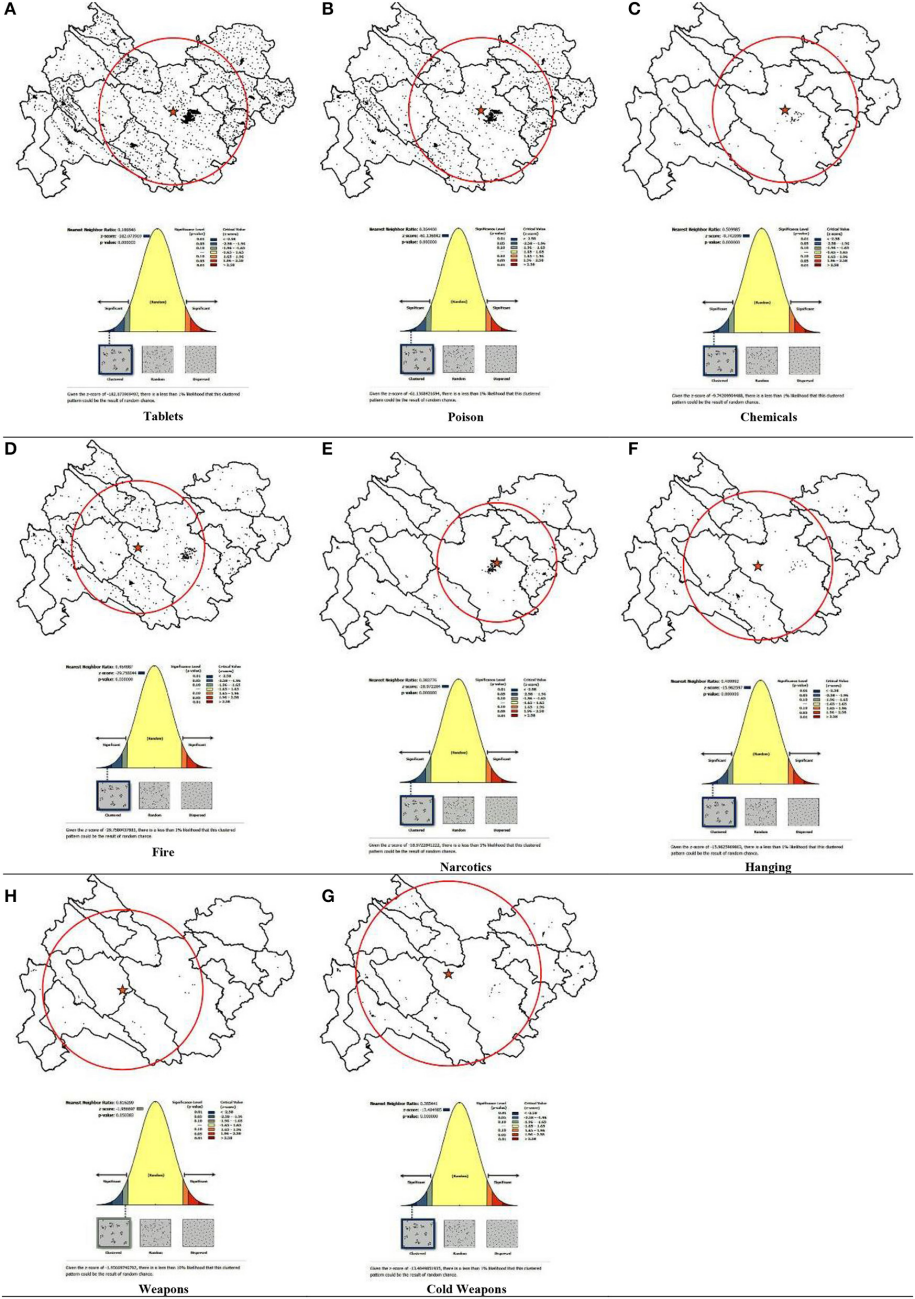
Geographic distributions (mean center and standard distance) and the analyzing patterns (average nearest neighbor). **(A)** Tablets, **(B)** Poison, **(C)** Chemicals, **(D)** Fire, **(E)** Narcotics, **(F)** Hanging, **(G)** Weapons, and **(H)** Cold weapons.

Figure 1 (average nearest neighbor) shows that methods of suicide attempts was clustered. Further, Nearest Neighbor Ratio for all methods were as follows: drug overdose (0.18, Z-score: −18.07), toxins and pesticides (0.36, Z-score: −61.13), chemical poisoning (0.50, Z-score: −9.74), self-immolation (0.46, Z-score: −29.75), narcotic drug poisoning (0.38, Z-score: −18.97), hanging (0.40, Z-score: −15.96), firearms (0.81, Z-score: −1.95), and self-mutilation (0.38, Z-score: −13.40), respectively.

## Discussion

This study examined the spatial patterns of suicide attempts among 18,331 people in Kermanshah province, western Iran. The findings of our study show that the spatial patterns of methods of suicide attempts including drug overdose, toxins and pesticides, chemical poisoning, self-immolation, narcotic drug poisoning, hanging, firearms, and self-mutilation in Kermanshah province were clustered. This finding was similar to the results of studies conducted in Ohio (1), and Australia (27). Some studies suggested that socio-economic deprivation, low household income, low level of education, lack of social support are effective in the formation of suicide clusters (27, 28). According to the cluster pattern of suicide attempts in Kermanshah province, it is suggested that this issue be further investigated in future studies.

On the other hand, the findings of this study show that residents of the central areas of Kermanshah province mostly used methods of drug overdose, toxins, and chemicals for suicide attempts. This situation is probably influenced by the proximity to the metropolis of Kermanshah and easier access to them. Results reveal the concentration of self-immolation, hanging, and firearms in the western regions of Kermanshah province. The easy access to firearms could be plausibly attributed to the Iraq-Iran war, and the western regions' proximity to the Iraqi border. The cluster pattern of self-immolation and hanging methods in these areas is likely influenced by rural culture and the diversity of different religions in this area of Kermanshah province. The spatial pattern of suicide attempts with narcotics in the eastern parts of Kermanshah province is probably due to the entry of drugs from the east of Kermanshah province and easier access to narcotics in these areas.

The most common method of suicide attempt in Kermanshah province was drug overdose, which was similar with the findings of (29), but different (30) from other studies conducted in Iran. The reason for the difference in suicide methods is probably due to different social harms that have multiple social roots. The results of other studies also attest that forces and pressures in society and social groups encourage or denounce suicide (14). Existing literature also indicate the role of other determinants such as accessibility and acceptability in the choice of suicide methods (31). The prevalence of suicide with drug overdose can be attributed to the availability of drugs, familiarity with various drugs and painlessness of the method. One of the issues that should be considered by the authorities is the statistics of self-immolation in Kermanshah province (more than 4%) considering its high fatality. The study of Ahmadi et al. (2008) also states that Iran has the highest rate of self-immolation in the world (11). This method of suicide is important because it has a cultural context and political influence in different countries (1). It seems that the use of this method has become a common phenomenon in Iran (11, 22, 24, 30). According to the liberal feminism, self-immolation is a terrible and frightening protest of women against the dominant view of patriarchy. Gender inequalities and the limited role of women in developing societies have led to their tendency toward self-immolation (32).

The results further reveal that drug overdose, poisoning by toxins and pesticides, and self-immolation were more common in women, while the use of firearms, self-mutilation and hanging were more evident methods in men. The results of other studies also show that the methods of drug overdose and poisoning were more common in women compared to firearms in men (31). In our study, men had used aggressive methods of suicide attempts compared to women, in line with other studies (30), which resulted in higher deaths due to suicide. Furthermore, a comparison of suicide attempts in age groups show that young people (16–25 years) had the highest rates in all methods. Existing research has shown that ages 15–29 years are high-risk age groups for suicidal behaviors (23). In this regard, India has high rate of youth suicide. In Indian society, it was a distinct phenomenon with various bio-psycho-social determinants (33).The World Health Organization (WHO) also identified suicide as the second leading cause of death in the age group of 15–29 years (34).

The drug overdose and pesticides poisoning were more common in singles while self-immolation and narcotic poisoning methods were more common in married people. Married used violent methods than single people, which can be due to marital conflicts, family problems and emotional failures (11). The findings of other studies in the United States were comparable to ours (35). Although Durkheim believed that marriage is a buffer for suicide, the methods of suicide attempts distributed according to marital status seems to be a complex issue and need further research.

The results on methods of suicide attempt according to the place of residence show that in all methods the percentage of urban people was higher except for firearms in which the rural people had the higher percentage. This finding was similar to an Australian study (36). Evidence from United States show that most suicides in Harris County occurred with firearms (37). In our study, the percentage of suicide attempts resulting in death was higher only in the use of firearms (51%). The results of other studies have shown that enacting laws and policies that reduce gun ownership can reduce suicide (37).

Suicide is one of the major public health problems across the world (1) and has recently imposed huge costs on communities and health care systems (2). Suicide attempt is a manifestation of one's suffering and has many negative effects on family, friends, and society (3).

## Conclusion

The results of the present study show that the methods of attempted suicide in Kermanshah province were clustered. Methods used to suicide attempt in the central areas of province were poisoning with drugs, pesticides and chemicals. In the western areas of the province, the common methods were self-immolation, hanging, and firearms, while the more evident method in the eastern areas was narcotics. The most common method of suicide attempts was drug overdose. Methods of suicide attempt with firearms, self-mutilation and hanging were more common in men, while drug overdose, poisoning by pesticides, and self-immolation were more common in women. The highest number of suicides was observed in the age group of 16–25 years and people with low education. Given that the methods of suicide attempt varied in different areas of province, it is suggested that different policies need to be considered to reduce people's access in various geographical parts of the province. It is also suggested that planning policies are developed in accordance with the location to reduce suicide attempts in Kermanshah province.

## Data availability statement

The original contributions presented in the study are included in the article/supplementary material, further inquiries can be directed to the corresponding author/s.

## Ethics statement

The studies involving human participants were reviewed and approved by the Ethics Committee of the Kermanshah University Medical Sciences. The patients/participants provided their written informed consent to participate in this study. Written informed consent was obtained from the individual(s) for the publication of any potentially identifiable images or data included in this article.

## Author contributions

NK, SS, and AZa were responsible for the study conceptualization and led the paper's writing. AA and AZa conducted the literature review and assisted in writing the paper. RT and SG performed the analysis, assisted in interpreting the data, and writing the paper. KY and SM assisted with the interpretation of the results and drafting programmatic implications. AZa and AZi were responsible for the data collection and coordination of the study. AZa co-led the conceptualization, supervised all aspects of writing the paper, and provided extensive comments on the manuscript. All authors were responsible for the study. All the authors have read and approved the final manuscript.

## Funding

The project received financial support from the Deputy Head of the Research and Technology Department of the Kermanshah University of Medical Sciences. The article processing cost is covered by the Kermanshah University of Medical Sciences, Kermanshah, Iran.

## Conflict of interest

The authors declare that the research was conducted in the absence of any commercial or financial relationships that could be construed as a potential conflict of interest.

## Publisher's note

All claims expressed in this article are solely those of the authors and do not necessarily represent those of their affiliated organizations, or those of the publisher, the editors and the reviewers. Any product that may be evaluated in this article, or claim that may be made by its manufacturer, is not guaranteed or endorsed by the publisher.
